# The COVID-19 pandemic reveals the wide-ranging role of biobanks

**DOI:** 10.3389/fpubh.2023.1256601

**Published:** 2023-09-01

**Authors:** Dovilė Juozapaitė, Mantas Minkauskas, Inga Laurinaitytė, Martyna Simutytė, Laimonas Griškevičius, Daniel Naumovas

**Affiliations:** ^1^Vilnius Santaros Klinikos Biobank, Hematology, Oncology and Transfusion Medicine Centre, Vilnius University Hospital Santaros Klinikos, Vilnius, Lithuania; ^2^Hematology, Oncology and Transfusion Medicine Centre, Vilnius University Hospital Santaros Klinikos, Vilnius, Lithuania

**Keywords:** COVID-19, pandemic, mitigation, management, biobank, hospital, samples, sequencing

## Abstract

The pandemic of COVID-19 reached an unprecedented scale in terms of spread and deaths, its mitigation required a joint effort of governments, hospitals, private companies and other organizations. One type of organization that could undertake a major role in the process is biobank – a mediator between clinical practice and research. Naturally, biobanks are well equipped to alleviate the burden of a pandemic with their expertise in biospecimen and health information collection, sample preparation and storage, bioethics and project management. Here, we present the participation of Vilnius Santaros Klinikos Biobank (BB VSK), Lithuania in the overall management of the pandemics on the national level. We further discuss the role of biobanks in preparation and management of future pandemics.

## 1. Introduction

SARS-CoV-2 virus appeared for the first time in late 2019 in Wuhan, China and did not take long to evolve into the worldwide coronavirus pandemic of 2019 (COVID-19) (1). As a result, hundreds of thousands of people have died in the first few months (2). In order to solve this, scientists and clinicians have started a number of research programs aimed at developing diagnostic tests, vaccines as well as identifying the best treatments (3, 4). In order to succeed, these projects require the analysis of biological samples obtained from negative and positive COVID-19 patients and discovery of relationship between test results, clinical and epidemiological data (5).

Biobanks are mediators between clinical practice and research and are responsible for biospecimen collection, processing, storage, and distribution as well as associated health information management (6). Key benefits provided by biobanks are existing collections of samples and data, extensive expertise in sample preparation, storage, bioethics, and project coordination. Vilnius Santaros Klinikos Biobank (BB VSK) is a hospital-based, non-profit biobank located in Vilnius, Lithuania. COVID-19 has affected Lithuania severely, with 344 deaths per 100,000 inhabitants. As of May 18th, 2023 the total death count reached 9,598 and 67% of the population has been fully vaccinated.

A number of problems caused by COVID-19 and other pandemics as well as possible mitigation actions have been proposed by other biobanks or biobanking organizations (7–11). Broadly and chronologically such challenges could be assigned to the following categories: management, diagnostics, treatment, vaccination, sequencing and surveillance. At BB VSK we have implemented a number of ideas which did help in fighting the pandemic at the hospital and national level. This article is meant to provide insights for hospital managers and other biobanks in preparation against future pandemics.

## 2. Results

### 2.1. Management

The COVID-19 pandemic posed major challenges to decision-makers in governments, hospitals, schools, and businesses. It has already been confirmed that delayed implementation of policies lead to higher mortality (12). Some researchers compare the management of the COVID-19 pandemic to the management of global warming and call both a “wicked problem.” Limited time, no clear central authority, lack of trust in governing institutions, and policies irrationally discounting the future are just a portion of issues the managers have to solve (13).

Following international tendencies, the Government of the Republic of Lithuania set up the Advisory Council of Independent Experts for COVID-19 management with regular meetings to discuss the most urgent problems (14). BB VSK proactively participated in several such meetings to provide recommendations about COVID-19 testing and surveillance guidelines. Also, our Biobank employees used their expertise and knowledge to review draft versions of regulations and resolutions.

In addition to collaboration with the Government, the Biobank provided the hospital management with updates on the most recent literature regarding diagnostics, virus biology, vaccination, or new emerging SARS-CoV-2 variants. To ensure that recommendations are not only theoretical but also practically confirmed and without delays, the decision to start biobanking samples from COVID-19 patients was made early in the pandemic (March 18, 2020) shortly after the first COVID-19 case in Lithuania (February 24, 2020). In total, 87,254 samples related to COVID-19 were collected and assigned to different collections, such as hospitalized for COVID-19, health care workers who underwent full vaccination course, a cohort of vaccinated immunocompromised patients, convalescent plasma donors. Technical storage recommendations were adapted from Centers for Disease Control and Prevention (CDC) guidelines (15).

Biobanks can only collect biological samples with an informed consent. Before the pandemic, it was a normal practice to have paper consent forms. However, it is known that SARS-CoV-2 can stay active on paper more than 3 h (16, 17) so to ensure the safety of hospital’s personnel and other patients BB VSK introduced e-consent infrastructure.

Regarding personnel, we at BB VSK also faced the challenge of a highly varied workload of processed samples per day. Besides the health status of employees themselves, this also depends a lot on pandemic severity and sample collection timepoints. In our experience, two of the most effective mitigation actions were: 1) cross-training of employees (also involving more experienced ones) and 2) maintaining a number of smaller administrative and/or less time-consuming projects which provided clear responsibilities for employees during the less intensive periods.

As the pandemic evolved so did the challenges the decision-makers had to face. The start of vaccination was an important milestone in the fight against SARS-CoV-2 but it required a specific infrastructure to be efficient. The storage temperature of the Pfizer-BioNTech COVID-19 vaccine is between −90°C and −60°C (18). Many hospitals did not have the infrastructure and experience to ensure secured, monitored storage and distribution of vaccines (19, 20). BB VSK was able to contribute ultra-low temperature freezers for vaccine storage and ensure good practices such as monitoring of temperature at all times, locked freezers, limited and traceable access to the storage room.

### 2.2. Diagnostics

After urgent sequencing of the first SARS-CoV-2 cases (21), PCR-based nucleic acid detection tests were adapted to detect the emerging virus. Such tests were a major achievement in the management of the pandemic. However, increased global need for reagents and consumables limited the testing capacities (22–24).

Lithuania tried to ensure a sufficient testing rate by purchasing rapid IgM and IgG antibody detection tests (AMP Rapid Test SARS-CoV-2 IgG/IgM, AMEDA Labordiagnostik GmbH, Austria). To give evidence-based recommendations for the application of such tests the scientists from BB VSK and Vilnius University Life Sciences Centre created the plan to evaluate the clinical sensitivity and specificity (25). Samples needed for the testing were obtained from BB VSK. In total 150 blood plasma samples collected before the pandemic and 50 blood plasma samples from COVID-19 PCR-positive patients were used to conclude that the IgM and IgG specificity was 100 and 100%, respectively, and IgM and IgG sensitivity for the early infection diagnostics was 10 and 60%, respectively. The results were reported to the Government and led to such recommendations: 1) the tests were sensitive and specific to detect IgG antibodies, 2) blood and plasma samples were equally suitable for such testing, and 3) these tests were not suitable for early detection of IgM as an indicator of COVID-19 infection.

Another challenge associated with extensive PCR testing is a limited throughput and a high price. Vilnius University Life Sciences Centre scientists decided to create group PCR for a higher throughput SARS-CoV-2 testing when separately collected nasopharyngeal swabs are combined in one reaction (26). BB VSK provided scientists with 75 SARS-CoV-2 positive and 275 SARS-CoV-2 negative nasopharyngeal swab samples. Researchers showed that the optimal pool size of five samples would significantly reduce the testing costs but retain the diagnostic sensitivity at >86% and suggested applying such method when testing a group of individuals with an expected positivity rate of <6.5%.

The testing of immunocompromised patients faced a specific challenge. Some patients with hematological malignancies and immunocompromised conditions were continuously positive using nucleic acid detection methods for several months making reactivation and reinfection diagnosis challenging. There was a need for a molecular tool that could distinguish the detection of residual viral RNA fragments from full-length viral RNA sequences and detect active viral replication. BB VSK worked together with Princeton University scientists to create and test the long-range PCR method (27). This method uses oligo(dT) primers for reverse transcription with the aim to get full-length transcripts that are later used in the qPCR assay to detect viral amplicons. The analysis showed that results using long-range RT-qPCR and regular PCR from 10 patients less than 5 days after the onset of symptoms were not significantly different. Contrarily, the analysis of samples collected more than 15 days after the onset of symptoms were significantly different when comparing both methods. This test complemented other diagnostic tools to detect active viral replication.

### 2.3. Treatment and vaccination strategies

Virus-specific T (VST) cells were reported to be active against fighting SARS-CoV-2 infections in immunocompromised patients (28, 29). A feasibility study using cells from 6 donors has also been carried out in Vilnius University Hospital Santaros Klinikos (unpublished results). BB VSK supported the research project with expertise in the manufacturing of VST products, quality control tests (immunophenotyping, IFNg secretion, cytotoxicity) and residual viable cell cryopreservation. The project aims to use the cells for more extensive analysis, e. g. investigation of activation and polyfunctionality biomarkers.

The COVID-19-related mortality in immunocompromised patients is significantly higher compared to the healthy population (30, 31) and the vaccine effectiveness in these patients was unclear, as they have not been included in the clinical trials (32, 33). To address this, BB VSK supported a large national prospective cohort study of RNA vaccines in hematological patients (34). In this project antibody responses to both the first and second BNT162b2 vaccine (Comirnaty, Pfizer-BioNTech) were investigated in 893 hematology patients and 68 healthy healthcare workers. The study found that a number of fully vaccinated patients with hematological malignancies remain at high risk of developing severe and fatal COVID-19 disease, emphasizing the importance of adherence to non-pharmacological interventions and household vaccination while SARS-CoV-2 is circulating in the community. In this project, BB VSK was involved in the study design and was responsible for sample processing and storage. As of May 2023, more than 26,000 samples from more than 1,400 patients and healthcare workers have been collected and processed into serum, plasma, lysed white blood or viable mononuclear cells. Also, a lesson learned was to standardize data input early on. During the biobanking process large amounts of data are handled, therefore, correcting the mistakes before each analysis or integration with other IT platforms results in a lot of wasted time. The hectic working environment during a pandemic is particularly prone to this mismanagement.

### 2.4. Monitoring of virus variants

Since the beginning of the pandemic in December 2019, hundreds of genetically distinct SARS-CoV-2 lineages have evolved (35). In some cases, the virus may mutate sufficiently to become undetectable by routine diagnostic tests, evade the immune system and complicate vaccine development or make the existing drugs ineffective (36). Virus genomic surveillance remains fundamental to understanding the evolution of the virus, the risk factors for severe disease and the impact of vaccination on public health. Country-wide, periodic genomic sequencing at the level of at least 0.5% of all positive cases helps to detect new circulating variants of SARS-CoV-2 (37).

To start a country-wide genomic surveillance sample and data infrastructure as well as sufficient financing resources should be provided. As long as biobanks are proficient in sample and data management, they are in a perfect position to facilitate the government to start a sequencing project. In the first year of the pandemic, only a small number of samples was sequenced in Lithuania. With the detection of the first SARS-CoV-2 variant of concern alpha (B.1.1.7) in the United Kingdom in December 2020 (38), BB VSK initiated a sequencing of the biobanked 1,000 COVID-19 samples. This was possible due to the collaboration with Thermo Fisher Scientific Baltics, Vilnius, Lithuania. The results showed that in Lithuania during the period of December 2020 and January 2021, the most prevalent lineage was B.1.177, which at first became prevalent in Spain and then spread across the rest of Europe (37). At that point, the alpha variant had not yet become widespread in Lithuania. The results of this study and the necessity of routine virus mutation surveillance were presented to the Government of Lithuania, which then financed the country-wide sequencing project. Six institutions contributed to this project. On average 12.5% of positive cases have been sequenced each month (approximately 2,600 samples per month) (39). All virus sequences have been deposited into the GISAID (40) database.

As a result of this project, it was possible to monitor when and what variants emerged in our country. A special case worth mentioning was the omicron variant and a drug named Ranapreve – a mix of two monoclonal antibodies. Omicron had mutations in exactly the same positions against which Ranapreve drug was designed, which made this drug completely ineffective in patients infected by omicron (41). In the winter of 2021–2022 it became necessary to identify different SARS-CoV-2 variants as fast as possible because there was an increase in COVID-19 cases caused by the omicron variant. Although sequencing is an accurate method to perform virus genomic surveillance, it is expensive, time consuming, requires special equipment and experts in bioinformatics. At best, sequencing results can be obtained in 2–3 days (42). PCR-based diagnostic approaches could help with many of those problems. Weeks after new variants of concern arose, commercial kits, detecting unique mutations, swiftly appeared in the market. Although these tests could differentiate SARS-CoV-2 variants, they were expensive and public hospitals could not obtain them as fast as they would like to because of the public procurement processes. This is the reason why hospitals could have scientific laboratories ready to develop in-house methods. In our hospital an in-house, multiplex PCR test that targeted specific mutations was adapted depending on the circulating variants and then implemented in clinical practice. In order to clinically validate this, biobanked samples and knowledge have been applied.

### 2.5. Surveillance

Surveillance testing within healthcare facilities and schools has a high potential to decrease the frequency of severe outbreaks of COVID-19 (43). However, daily, bi-weekly, or even weekly testing of a healthy population is an expensive and burdensome procedure (44). Strategies to monitor the prevalence of the virus in wastewater and to detect asymptomatic cases in schools, workplaces or hospitals have already been described (45, 46). Our biobank initiated hospital surface swab testing. The testing area was divided into different zones specific to different personnel groups. Each zone had different objects that were touched multiple times during the day by the workers, for example, a computer keyboard or a door handle. Such objects were swabbed at the end of each day and pooled with other swabs from the same zone. In case of a positive swab, a specific personnel group would be tested to find the person who left viral particles on the surface. In total, 5 screenings of 15 different rooms were performed. Positive control rooms – COVID-19 wards. During the screening process, 1 room was tested positive with high Ct value (Ct(ORF1ab) = 36) but after testing personnel working in the room we were not able to find the SARS-COV-2 positive person. There were also 3 incidents where the surface PCR result was negative but employees were tested positive. Overall, results showed that it is possible to detect SARS-CoV-2 RNA on the surface. However, disinfection of most hospital surfaces was so frequent, that this method became inexpedient. On the other hand, the method could be suggested for other, non-healthcare, organizations, where the disinfection regimes are less thorough. We have also investigated an alternative approach to prevent outbreaks – PCR test from swabbed masks. Compared to surface PCR disinfection was not a limiting factor. However, reproducibility of this method was poor. We suggest this might be due to 1) different manufacturers’ masks having different RNA isolation or PCR inhibitors, 2) the protocol of sampling being difficult to manage in practice.

A cheaper and less invasive surveillance method was still needed. One way to make the tests cheaper is to pool samples, but nasopharyngeal sample collection is still an unpleasant invasive process. Therefore, BB VSK suggested using the nasal pooling PCR method. It was already demonstrated that SARS-CoV-2 testing from nasal swabs can produce comparable results to nasopharyngeal swabs (47).

**Figure 1:**
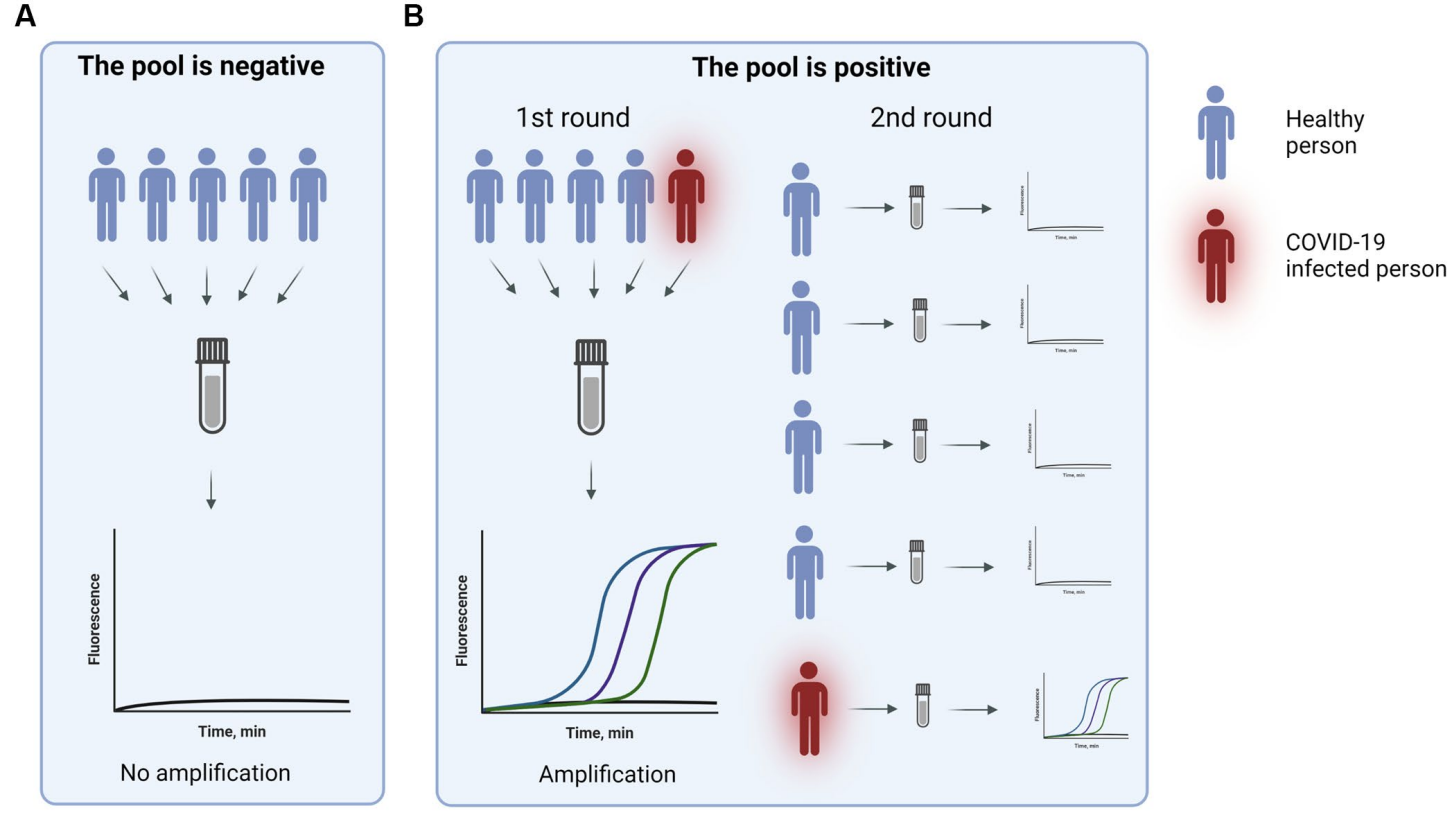
COVID-19 sample pool testing.

To confirm that pooled samples can provide high sensitivity a study to compare pooled nasal swabs to individual nasal swabs was performed. In the testing setting pools of 5–7 swabs were used. To validate the method two types of pools were used: 1) negative pool - all individual swabs were negative, 2) positive pool – one individual swab was positive. In total, 51 pools were tested out of which 22 pools were positive and 29 were negative. The demonstrated sensitivity was 96%. For more than 2 years this method was used for healthcare workers and patient surveillance in departments most vulnerable to COVID-19 (Figure 1).

Our validated pool nasal swab assay was also presented to the Vilnius city municipality, which then implemented this test in routine school surveillance testing. When the testing became mandatory, it was suggested to reopen the schools in severely affected municipalities (48).

### 2.6. Timeline

**Figure 2:**
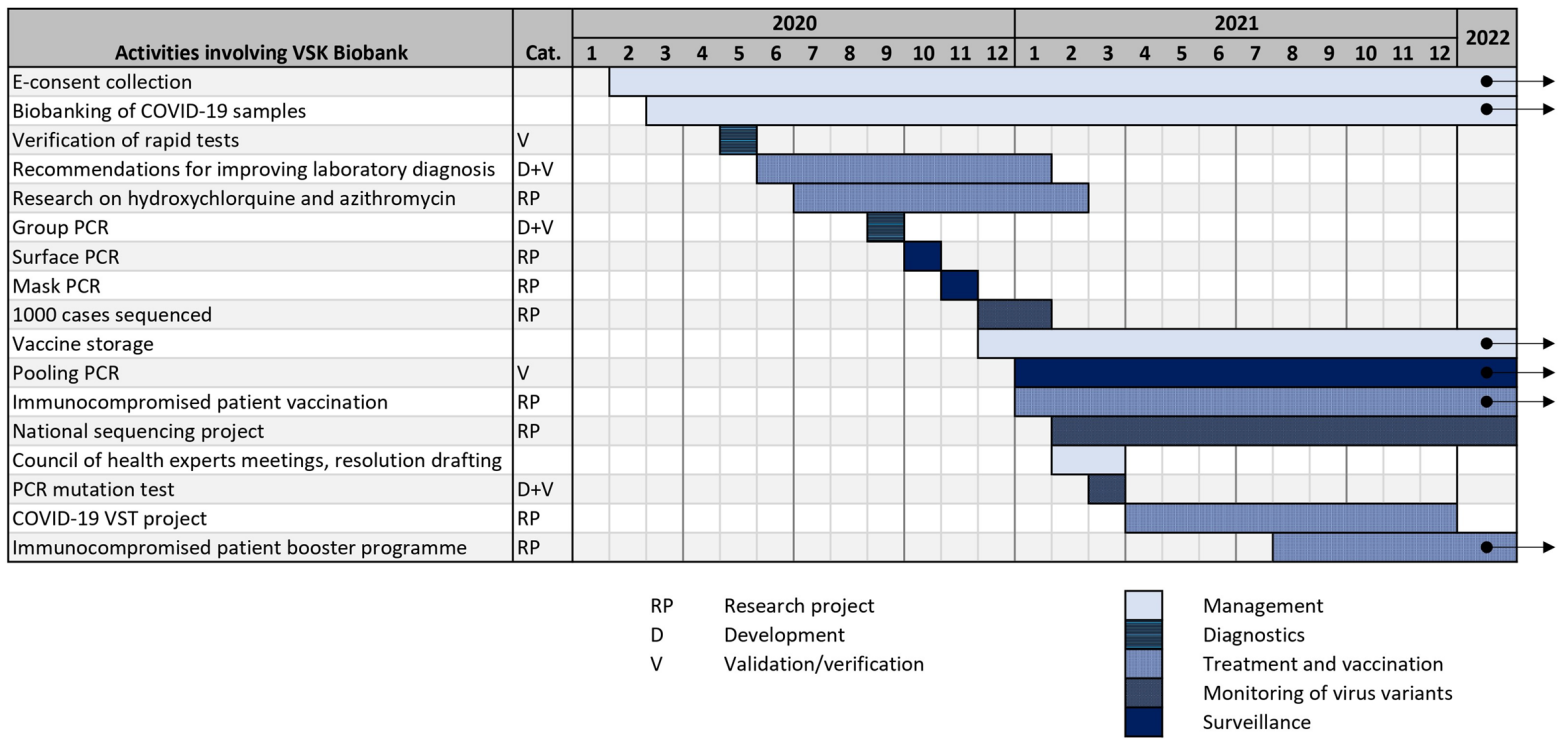
Gantt chart of BB VSK projects related to COVID-19.

All of the aforementioned BB VSK projects, that are related to COVID-19 pandemic, are summarized in timeline in Figure 2. As mentioned before, biobanks can help in fighting pandemics using a variety of strategies: management, diagnostics, surveillance, sequencing, vaccination and treatment. It is crucial that some strategic infrastructural decisions are made early in the pandemic and should be revisited during the course of it. The lack of diagnostic assays in the beginning of the pandemic encourages biobanks to get proactively involved in the development and validation of such assays. With growing numbers of infected people, different surveillance programs can be implemented, like pooling PCR. Data and samples collected in the biobank can be used to determine the best treatment and vaccination algorithms which are relevant during the whole timeline of the pandemic and also afterwards. Surge of virus variants with altered characteristics leads to increased need for monitoring of variants.

## 3. Recommendations for biobanks

1. Collect a set of samples during non-pandemic times. These different source samples will serve as negative controls. The processing and storage methods should be chosen with future applications in mind.
2. Use electronic consent forms if possible.
3. Follow general news about emerging viruses and the epidemiological status of existing ones and share expert opinion with decision-makers.
4. Follow the recommendations by WHO, ECDC, BBMRI-ERIC, and ISBER on sample storage.
5. Create and follow data standardization practices despite chaotic and rushed operations during the pandemic.
6. Continue storing at least a minimal number of new samples during the course of the pandemic. In that way, most of the virus variants will be stored.
7. Support vaccination programs with the pre-existing infrastructure and best practices regarding storage.
8. Employ at least one molecular biologist, who could develop and validate home-made diagnostic assays.
9. Consider different approaches to manage large variations of day-to-day workload and human resources in a sample processing laboratory.
10. Actively communicate with the scientists and physicians about collections stored in a biobank - biobanks can provide great value by enabling cooperation when the time is precious.

## Data availability statement

Publicly available datasets were analyzed in this study. This data can be found at https://gisaid.org/.

## Ethics statement

The studies involving human participants were reviewed and approved by Vilnius Regional Bioethics Committee, Faculty of Medicine, Vilnius University, M. K. Čiurlionio g. 21, LT-03101, Vilnius. The patients/participants provided their written informed consent to participate in this study.

## Author contributions

DJ: Conceptualization, Data curation, Investigation, Project administration, Visualization, Writing – original draft, Writing – review & editing. MM: Data curation, Investigation, Visualization, Writing – original draft, Writing – review & editing. IL: Methodology, Writing – review & editing. MS: Methodology, Writing – review & editing. LG: Conceptualization, Writing – review & editing. DN: Supervision, Writing – original draft, Writing – review & editing.

## Funding

The author(s) declare financial support was received for the research, authorship, and/or publication of this article.

VST project has been financed by the Research Council of Lithuania, financing agreement No. S-DNR-20-12. Publication fees are funded by Vilnius University Hospital Santaros Klinikos.

## Conflict of interest

The authors declare that the research was conducted in the absence of any commercial or financial relationships that could be construed as a potential conflict of interest.

## Publisher’s note

All claims expressed in this article are solely those of the authors and do not necessarily represent those of their affiliated organizations, or those of the publisher, the editors and the reviewers. Any product that may be evaluated in this article, or claim that may be made by its manufacturer, is not guaranteed or endorsed by the publisher.
